# Bacteria Floc, but Do They Flock? Insights from Population Interaction Models of Quorum Sensing

**DOI:** 10.1128/mBio.00972-19

**Published:** 2019-05-28

**Authors:** Hana Ueda, Kristina Stephens, Konstantina Trivisa, William E. Bentley

**Affiliations:** a Department of Mathematics, University of Maryland College Park, College Park, Maryland, USA; b Graduate Program in Applied Mathematics & Statistics, and Scientific Computation, University of Maryland College Park, College Park, Maryland, USA; c Fischell Department of Bioengineering, University of Maryland College Park, College Park, Maryland, USA; d Institute for Bioscience and Biotechnology Research, University of Maryland, College Park, Maryland, USA; Korea Advanced Institute of Science and Technology; University of California, Berkeley; Rensselaer Polytechnic Institute

**Keywords:** chemotaxis, flocking, quorum sensing

## Abstract

Quorum sensing (QS) enables coordinated, population-wide behavior. QS-active bacteria “communicate” their number density using autoinducers which they synthesize, collect, and interpret. Tangentially, chemotactic bacteria migrate, seeking out nutrients and other molecules. It has long been hypothesized that bacterial behaviors, such as chemotaxis, were the primordial progenitors of complex behaviors of higher-order organisms. Recently, QS was linked to chemotaxis, yet the notion that these behaviors can together contribute to higher-order behaviors has not been shown. Here, we mathematically link flocking behavior, commonly observed in fish and birds, to bacterial chemotaxis and QS by constructing a phenomenological model of population-scale QS-mediated phenomena. Specifically, we recast a previously developed mathematical model of flocking and found that simulated bacterial behaviors aligned well with well-known QS behaviors. This relatively simple system of ordinary differential equations affords analytical analysis of asymptotic behavior and describes cell position and velocity, QS-mediated protein expression, and the surrounding concentrations of an autoinducer. Further, heuristic explorations of the model revealed that the emergence of “migratory” subpopulations occurs only when chemotaxis is directly linked to QS. That is, behaviors were simulated when chemotaxis was coupled to QS and when not. When coupled, the bacterial flocking model predicts the formation of two distinct groups of cells migrating at different speeds in their journey toward an attractant. This is qualitatively similar to phenomena spotted in our Escherichia coli chemotaxis experiments as well as in analogous work observed over 50 years ago.

## Abstract (precis)

Our modeling efforts show how cell density can affect chemotaxis; they help to explain the roots of subgroup formation in bacterial populations. Our work also reinforces the notion that bacterial mechanisms are at times exhibited in higher-order organisms.

## INTRODUCTION

Quorum sensing (QS) is a form of communication that bacteria employ to coordinate behavior in a collective manner. The means of communication, or collective gene regulation, is accomplished through chemical signals called autoinducers. By producing and collecting autoinducers, bacteria such as Escherichia coli, Vibrio fischeri, and Pseudomonas aeruginosa can determine the density of cells in their surroundings. For one family of autoinducers (acyl homoserine lactones, AI-1), the autoinducers are species specific so that the enumeration of cell density reflects self. For another family (linear 4,5 dihydroxy 2,3 pentanedione [DPD] and ring-formed enantiomers, AI-2), the autoinducer is secreted by many strains but recognized in different ways (1) by different strains (2), enabling microbial recognition as well as enumeration of cell density. In both systems, an increase in cell density increases the surrounding concentration of autoinducers, which, in turn, triggers a series of signal transduction mechanisms. This facilitates changes in protein expression, among other phenotypes. Hence, when there is a sufficient number of bacteria (a quorum) in a specific locale, they collectively exhibit various phenotypes, such as bioluminescence, biofilm formation, and virulence factor expression, to name a few (2–4).

One approach to modeling these systems involves differential equations that represent specific genetic and biochemical mechanisms for autoinducer synthesis, uptake, and receptor binding, the transcription and translation from gene to protein of QS-related genes, and interactions of the various enzymes (e.g., phosphorylation) that allow for these processes to occur (1, 5–20). Even comprehensive mechanistic two-dimensional (2D)-agent-based models that delineate behavior as a function of the specific autoinducer’s signal transduction modality have appeared (1). In this work, we propose a different approach in which we model the general behaviors of species dynamics. By not emphasizing the genetic regulatory mechanisms, we focus solely on the more general picture pertaining to quorum sensing and behaviors that can be governed by quorum sensing, such as chemotaxis. That is, we draw on lessons from nature, where many species in the animal kingdom coordinate sensors, pattern recognition, and even population-scale behavior in an amazingly beautiful fashion that evokes both awe and mimicry.

“Flocking” describes the movement of biological entities that conforms to the information from the entities’ surroundings to adjust their actions, forming a collective motion. Collective motion, in turn, is observed among complex organisms, such as birds, fish, and even wildebeest (21–23); it has also been postulated to occur among bacteria (24). To quantify this emergent behavior and to further study the self-organizing dynamics of biological systems, Cucker and Smale introduced a dynamical model that analyzes these movements using population interactions (25, 26). Their representation of flocking was based on the distances between neighboring birds and weighted averages of their velocities to represent interactions. Specifically, with few parameters, the speed by which a bird adjusts its velocity can be made more, or less, a function of its nearest neighbors. A highly “collective” flock will closely regulate each bird’s velocity based on its neighbors and a more loosely controlled flock will consist of birds that only minimally tie their velocities to those of their neighbors. When each bird in a flock adjusts its speed in this way, every bird ultimately attains the same velocity. While the biological coordination of bird flocking has been attributed to sight (27) and mathematically expanded upon by Ha and Tadmor (28), the Cucker-Smale model is as follows:

$$\frac{d}{\mathrm{dt}}{\text{x}}_{i}\left(t\right)={\text{v}}_{i}\left(t\right)$$

and

$$\frac{d}{\mathrm{dt}}{\text{v}}_{i}\left(t\right)=\frac{\lambda }{N}\underset{1\leq j\leq N}{\sum }k\left[{\text{x}}_{i}\left(t\right),{\text{x}}_{j}\left(t\right)\right]\left[{\text{v}}_{j}\left(t\right)-{\text{v}}_{i}\left(t\right)\right].$$

The model states that the velocity of each bird, *i*, represented by the state variable *v_i_*(*t*), changes based on the velocities of the surrounding birds, *j*, with the symmetric weights *k*[*x_i_*(*t*), *x_j_*(*t*)] determining the strength of influence of the neighboring birds. *x_i_*(*t*) and *x_j_*(*t*) represent the positions of birds *i* and *j*, and *k*[*x_i_*(*t*), *x_j_*(*t*)] is a function of the distances between the birds. That is, *k*[*x_i_*(*t*), *x_j_*(*t*)] = *k*(|*x_i_* − *x_j_*|). *N* is the total number of birds, and λ is a factor controlling the strength of the flocking term. From this differential equation, Cucker and Smale proved that every bird approaches the same velocity, the behavior commonly observed among flocking birds. While chemotaxis provides a means for population-based behavior, there have been few reports attempting to ascribe such population-based movement to bacterial quorum sensing (29–35). Further, while the genetic basis for chemotactic behavior has been elucidated, and particularly well for E. coli (our model system), there have been no reports suggesting that QS is a bacterial analog of flocking commonly observed among higher-order organisms.

The mathematical definition of flocking provided in the work of Motsch and Tadmor (36) is given as definition 1: given a particle model with distances and velocities of each cell, *i*, represented by [*x_i_*(*t*), *v_i_*(*t*)]_*i* = 1,…,*N*_, the system converges to a flock provided that the following properties hold (where “max” is maximum and “sup” is superior): ${\text{lim}}_{t\to \infty }\;{\text{max}}_{i,}{}_{j}|v_{j}\left(t\right)-v_{i}\left(t\right)|=0$ and $\sup_{t\geq \infty }\;\max_{i}{}_{,j}|x_{j}\left(t\right)-x_{i}\left(t\right)|<\infty .$ In order to qualify as flocking, the distances must remain bounded and the velocities must asymptotically converge to the same value (for propositions and their proofs, see Text S1 in the supplemental material).

10.1128/mBio.00972-19.1 TEXT S1 Propositions and asymptotic analysis. Download Text S1, DOCX file, 2.1 MB. Copyright © 2019 Ueda et al. 2019 Ueda et al. This content is distributed under the terms of the Creative Commons Attribution 4.0 International license.

As for applications of flocking to cells, Di Costanzo et al. (37) incorporated this in modeling the motility of cells during morphogenesis of the posterior lateral line primordium in zebrafish. Ha and Levy (38) incorporated flocking when modeling the phototaxis of the cyanobacterium *Synechocystis* sp. In this paper, we build on the work of both Ha and Levy (38) and Ha and Tadmor (28). Our model introduces flocking not just as a method of representing velocities but as a way of representing quorum-sensing behavior. To do this, we have expanded the number of state variables of interest and tied them to the autoinducing molecular signal and its actuation of protein expression. By averaging autoinducer-mediated protein expression in a way similar to the way velocities are averaged and by using weighted terms to model the synthesis and uptake of autoinducers, positive- and negative-feedback behaviors can be accommodated. Further, we apply the concept of taking weighted averages to describe the dynamics in bacterial communication in tandem with chemotaxis. That is, the model takes into account the fluctuations in both cell velocity and cell protein expression as a result of changing autoinducer concentrations in the surrounding medium. The model that we present can be applied to any quorum-sensing species that (i) demonstrates positive and/or negative feedback and (ii) produces and collects autoinducers via diffusion (e.g., AI-1) or potentially active transport (e.g., AI-2).

**FIG 1:**
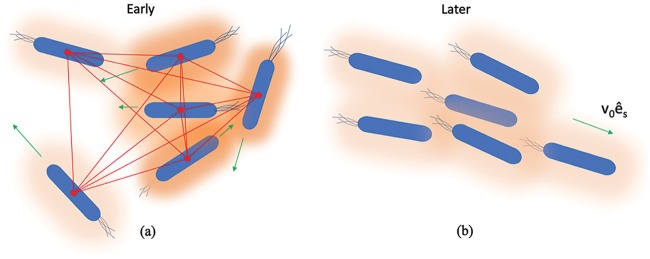
Emergent behavior based on neighbors. (a) Initially, cells with randomly distributed positions and of random velocity are exposed to a bacterial autoinducer at different concentrations (orange represents autoinducer concentration; proximal cells experience a higher concentration). In the model, the distances between cells and the values of flocking model parameters guide the progression in cell movement toward a collective uniform trajectory (*v*_0_*ê_s_*), as in panel b, where later, cells are aligned based on the progression of interactions between neighbors and prevailing autoinducer levels.

Quorum-sensing systems vary from species to species, utilize different kinds of autoinducers and different signal transduction motifs, and yield different emergent phenotypes or behaviors. There are many variations, and importantly, not all mechanisms are known. By incorporating the Cucker-Smale flocking terms and similar weighted terms, our goal is to create a relatively simple model that globally describes these quorum-sensing behaviors. The mathematical formulism is represented in Fig. 1, wherein, initially, individual cells are depicted at various distances from each other (indicated by connecting lines in Fig. 1a), with various velocities (indicated by arrows). The concentration of autoinducer is a function of cell number and cell-cell distance (concentrations are suggested by the intensity of the orange shadows). That is, an individual cell’s QS-mediated protein expression level is affected in the same way as its velocity. The cells indicated at the right (Fig. 1b) represent a population at a later time, when they have adjusted their velocities and protein levels and are close to an asymptotic alignment. Note that the terminal velocity attained is *v*_0_*ê_s_*. In this work, we postulated the following hypothesis: quorum sensing represents a bacterial analog to flocking, and as such, it can be similarly described mathematically, and its mechanisms might ultimately provide a means for understanding emergent and collective behaviors among higher-order species. To do this, we simulated the model in two ways: one allows QS-mediated protein expression to be directly coupled to velocity, and the other does not. We then compared model results of interesting experiments, including some of the earliest chemotaxis experiments reported over 50 years ago showing disparate bands of cells emerging from “home” in search of nutrients (39). We also qualitatively compared the results of our own previous experiments, which tested directly the bacterial search for new environs (35).

## RESULTS

### Uncoupled model.

We introduce a general model that focuses on the following four aspects of each cell: (i) *x_i_*, the position of cell *i*; (ii) *v_i_*, the velocity of cell *i*; (iii) *p_i_*, the protein expression of cell *i*; and (iv) *A_i_*, the concentration of autoinducers surrounding cell *i*. The model is a system of 4*N* ordinary differential equations that tracks the dynamics of the four above-named state variables. *N* represents the total number of cells, as follows:

(1) $$\frac{dx_{i}}{\mathrm{dt}}=v_{i}$$

(2) dvidt=λ1N∑j=1Nk1(xi,xj)(vj−vi)+F0(υoe^s−vi)

(3) $$\frac{dp_{i}}{\mathrm{dt}}=\frac{\lambda_{2}}{N}\overset{N}{\underset{j=1}{\sum }}k_{2}\left(x_{i},x_{j}\right)\left(p_{j}-p_{i}\right)+L_{0}\left(p_{\infty }-p_{i}\right)\psi_{A}\left(A_{i}\right)$$

(4) $$\frac{dA_{i}}{\mathrm{dt}}=\lambda_{3}\overset{N}{\underset{j=1}{\sum }}k_{3}\left(x_{i},x_{j}\right)r-k_{u}\left(x_{i}\right)A_{i}-k_{d}A_{i}$$

with the corresponding weights,

$$k_{1}\left(x_{i},x_{j}\right)=k_{2}\left(x_{i},x_{j}\right)=k_{3}\left(x_{i},x_{j}\right)=\frac{1}{{\left(1+{\left|x_{j}-x_{i}\right|}^{2}\right)}^{\beta }}$$

logistic function,

$$\psi_{A}\left(A_{i}\right)=\frac{1}{{\left[1+e^{c_{0}\left(c_{1}-c_{A}A_{i}\right)}\right]}^{a}}$$

and uptake function

(5) $$k_{u}\left(x_{i}\right)=a_{1}\exp\left(\frac{-{\left\{a\left[\gamma_{1}\left({\sum_{j=1}^{N}\left|x_{j}-x_{i}\right|}^{2\beta }\right)\right]-b\right\}}^{2}}{2c}\right)+a_{2}$$

This model is referred to as “uncoupled” because the protein expression equation (equation 3) is not coupled with the velocity equation (equation 4) (i.e., protein expression does not influence movement), unlike in the coupled model, which will be discussed later.

The weight functions *k*_1_, *k*_2_, and *k*_3_ are symmetric, which will prove useful in the subsequent asymptotic analysis. These functions decrease as the distance between the cells increases. The exponent in the denominator (β) controls how fast the functions decrease from 1. Qualitatively, β represents the scope of influence that the neighboring cells have on the individual. So, low β values (such as β = 0) would model a system in which the individual is influenced by all cells.

The logistic function Ψ*_A_*(*A_i_*) acts as a smoothed piecewise constant function and takes values ranging from 0 to 1 depending on the surrounding autoinducer concentration (*A_i_*) of cell *i*. The uptake function is modeled as a shifted Gaussian. *k_u_*(*x_i_*) is constructed in such a way so that the uptake rate increases as the density increases until reaching a maximum. Thereafter, the uptake decreases when the density is high. This is to reflect a decrease in metabolic activity caused by overcrowding (i.e., decreased nutrient availability when cells are crowded). With this Gaussian, we assume that there is a density range in which uptake is highest.

Equation 1 provides the definition of velocity. Equation 2 models the changing dynamics of the velocity of cell *i* by employing a flocking (population interaction) term and a source term. The flocking term addresses how the velocities of neighboring cells influence the individual's velocity. The weight function *k*_1_ as well as the parameter β determines how much influence that the other cells, *j*, have on cell *i*. Next, the source term models chemotaxing behavior in which the cell runs toward the higher concentrations of chemoattractant at a velocity of *v_o_ê_s_*, where *ê_s_* represents the unit direction vector. The strength of this term is controlled by the constant *F*_0_. The combination of both the flocking term and the source term generalizes the movements of these cells in response to the chemical stimuli. Note that depending on *F*_0_, the source term influences the movement of cells much more than the flocking term. In this way, a random perturbation by one individual, a small collection, or a perturbation that reflects an environmental cue can lead to global directional changes for the population, and these may accurately reflect natural circumstances.

Equation 3 tracks the dynamics in autoinducer-triggered protein expression, which we use as a marker of the collective output of the QS phenotype, such as biofilm formation (40) or agglomeration (32). To motivate the existence of equation 3, for the sake of a concrete example, we use the expression of bioluminescence by Vibrio fischeri (41). In this species, the autoinducer AI-1 (an acyl-homoserine lactone [AHL]) controls the regulatory system (LuxI/LuxR) that governs the production of both the autoinducer and various proteins. LuxI is an AI-1 synthase, and LuxR is an activator regulating transcription of the *luxCDABEG* operon. The *luxCDABEG* genes code for the regulation of bioluminescence, including the expression of the luciferase enzymes that are responsible for light emission. The flocking term then serves to average the bioluminescence of the surrounding cells. The additional term, which we call the source term, represents the bioluminescence triggered when the surrounding concentration of autoinducers passes a certain threshold. Above this threshold, the logistic function Ψ*_A_* approaches 1, thereby activating this source term. The strength of this term is controlled by the constant *L*_0_. In our model, flocking plays only a small part in the cell dynamics when the source term is in effect (i.e., the logistic function is not zero). That is, flocking behavior is more pronounced before the source term is activated.

The changes in the surrounding concentration of autoinducers of a particular cell are represented by equation 4. The equation is constructed as follows:

$$\begin{array}{l} \frac{dA_{i}}{\mathrm{dt}}=\text{synthesis}-\text{uptakes}-\text{sloughing off/degradation of autoinducers}\\ =\lambda_{3}\overset{N}{\underset{j=1}{\sum }}k_{3}\left(x_{i},x_{j}\right)r-k_{u}\left(x_{i}\right)A_{i}-k_{d}A_{i} \end{array}$$

and this is phenomenologically similar to several previous models (7, 20).

### Uncoupled model: simulations and discussion.

The model presented was constructed to analyze both the movement of cells toward a chemoattractant and autoinducer-triggered protein expression. To recap, this system consists of 4*N* differential equations, in which the four state variables, namely, position, velocity, protein expression, and surrounding autoinducer concentration, are assigned to each cell. *N* represents the total number of cells. The equation governing the surrounding concentration of autoinducer of cell *i* consists of a weighted synthesis term, an uptake term, and a degradation term. The uptake function can be customized to reflect the system. We initially used a logistic uptake function that was dependent on time (see property iv in Text S1 in the supplemental material (“Asymptotic Analysis”) to prove that the surrounding concentration of autoinducers converged to a constant value, which differs for each cell. Our assumption was that the cells grow sufficiently dense over time and that the uptake increases concomitantly and remains steady at an elevated level. The purpose of this logistic function was to find a simple representation of this general behavior that was with respect to time, instead of position, so that one could perform the asymptotic analysis of the differential equation.

**FIG 2:**
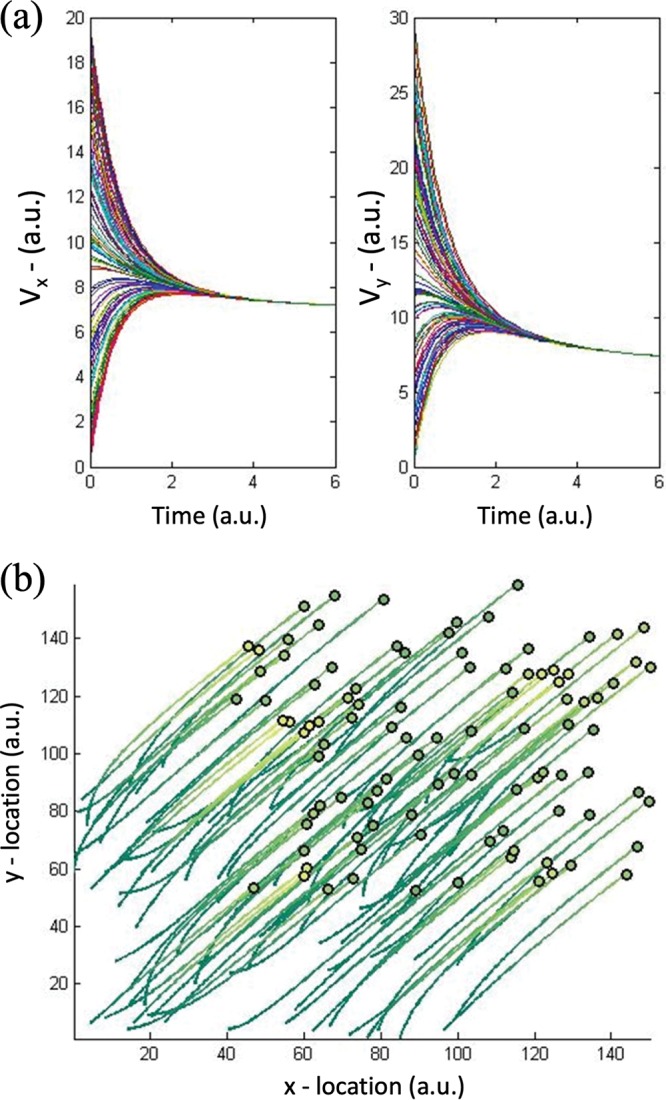
Simulated trajectories of a randomized initial population. (a) Cells exhibit a convergence of velocity in the *x* direction (*V_x_*) and *y* direction (*V_y_*), with λ_1_equal to 5 and β equal to 0.2 for 100 cells and with initial conditions randomly generated from a uniform distribution on the interval ([0, 20] and [0, 30] for *V_x_* and *V_y_*, respectively). (b) The cells are represented by circles. The trails represent the cell trajectories, and the emergence of yellow from green represents the emergence of QS-mediated gene expression. Note that the velocities of cells remain bounded (shown here for a fixed time [the proof is in Text S1 in the supplemental material]). a.u., arbitrary units.

In Fig. 2, we show how a randomized distribution of cells with randomized initial velocities ultimately converges toward a uniform velocity trajectory. In the plot, panel a reveals the rapid alignment of velocities toward the value *v_o_ê_s_* as time progresses. The velocities are parsed into x and y directions. Panel b includes trajectories of 100 cells in a 2D plane, the color indicating the level of protein expression for each cell, and this shifts from green to yellow as the QS-mediated activity is actuated. The final position in each trajectory is indicated by a dot, the color of which indicates the extent to which QS-actuated protein was accumulated. This simulation is purely a representative case, with constants indicated in the figure caption; it indicates generally the propensity by which the cells reached an asymptotic velocity and exhibited QS-mediated gene expression.

**FIG 3:**
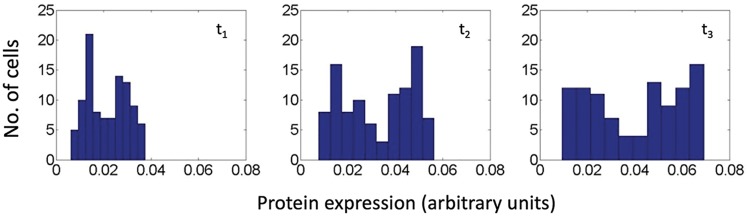
Histogram of QS-mediated protein expression in 100 cells. The logistic function Ψ*_A_*(*A_i_*) and the Gaussian uptake expression in the model enable the bimodal expression variation that is often observed for AI-2-mediated QS. The histograms depicted reveal a simulated bimodal distribution at three arbitrary time points (*t*_3_ > *t*_2_ > *t*_1_). Parameter values for the Gaussian uptake function are as follows: *a* equals 4, *b* equals 4, and *c* equals 27.81, with β equal to 0.62 and γ equal to 1/2,000.

For these simulations (***x_i_*** in **ℝ**^2^, ***v_i_*** in **ℝ**^2^, ***p_i_*** in **ℝ**^2^, and ***A_i_*** in **ℝ**^2^), we used a shifted Gaussian for the uptake function in which, at low cell densities, the uptake is low (the lowest being *a*_2_). At higher densities, the uptake increases. At even higher densities, the uptake begins to decrease. The reason for this is to account for the effect of overcrowding (e.g., nutrient depletion) on metabolic processes. Also, this allows for more interesting dynamics in the surrounding concentration of autoinducers, which can lead to a higher variance in protein expression among the population, as seen in Fig. 3, where the emergence of subpopulations of cells can be observed. This is more representative of AI-2-mediated QS, wherein there is a negative-feedback loop governing AI-2-mediated protein expression (1). A monotonically increasing uptake function with decreased distance would not result in this behavior, and this would be more representative of AI-1-mediated QS. Note also, however, that the variation in protein expression here is also attributed to the logistic function Ψ*_A_*(*A_i_*), which, together with the autoinducer concentration, governs the per-cell protein expression in the absence of neighbors.

**FIG 4:**
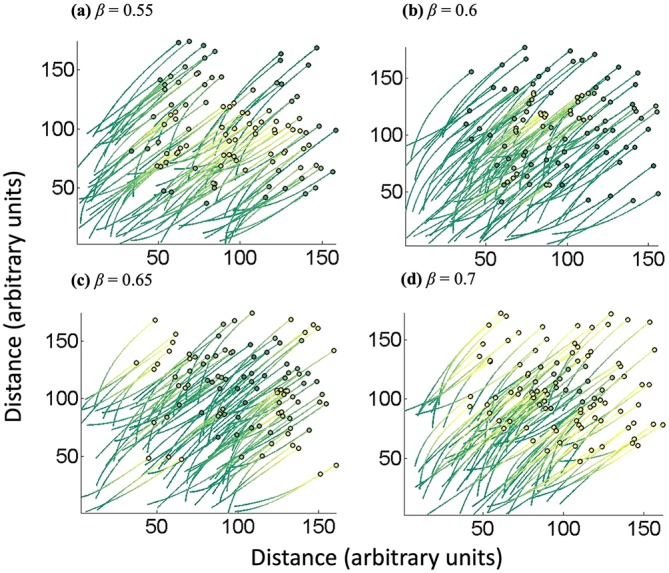
Trajectories of 100 cells initially randomly distributed but with different dependencies on their nearest neighbors. (a to d) Cell paths for a β of 0.55, 0.6, 0.65, and 0.7, respectively. The circles represent the cells, and the lines represent the paths that the cells have travelled (from time zero). The colors represent levels of QS-mediated protein expression (green is low, and yellow is high). At low β values, cells that are closer together are seen to be more yellow; they express more protein as a function of the autoinducer uptake. As the β value increases, however, there is a reversal in behavior. The cells which are at a higher density (middle of plots) express less protein than the cells at a lower density (on the periphery). This functional flexibility in the simulation is due to the Gaussian uptake term, in which the β value decreases the importance of the sum of the distances between cells. The cases observed in natural settings are more aligned with lower β values (e.g., a β of 0.55 or 0.6).

The β parameter in the model can be interpreted as the weight placed on the behaviors of the neighbors. When β is equal to 0, the individual cell places equal weight on all other cells. As β increases, the individual cares less about the behaviors of the cells further away. To illustrate, we varied β and illustrate resultant behaviors in order to qualitatively hone in on obtaining realistic β values. This type of simulation also illustrates the ease with which population-based behavior can be modified by simple adjustment of one parameter, β. In Fig. 4, the β values analyzed were 0.55, 0.6, 0.65, and 0.7. For the lower values of β (i.e., 0.55 and 0.6) (Fig. 4a and b), the cells at a higher density (those closer together and not on the periphery) express more protein (they are more yellow) than the cells at a lower density. That is, the cells at lower density are seen on the outskirts of the group, and these are all green, as are their entire trajectories. This is particularly well visualized in Fig. 4a, where the plot’s center is predominantly yellow, the color that is the visual sum of the colors of the individual trajectories that at times intersect. In Fig. 4b, the QS-mediated yellow is a bit more focused in the middle of the plot. This heuristic graph represents the prototypical QS response wherein the cells that are closer to others and experience the highest levels of autoinducer exhibit the greatest QS genetic response. As noted above, this is easily described by parameter β and the Gaussian shape of the uptake function, which is determined by the constants *a*, *b*, *c*, and *a*_2_, providing the range of distances between cells over which the autoinducer uptake is highest. Note that as β increases to 0.65 and 0.7 (Fig. 4c and d), the simulated result is largely reversed; cells at a lower density are observed to express more protein, which is counterintuitive in QS. This too is due to the Gaussian uptake function. As β is increased further, the cells with a greater difference from other cells (i.e., large |*x_i_*(*t*) – *x_j_*(*t*)|) are less influential on each other and, importantly, become more individualistic in their phenotype. Also, numerically, the autoinducer uptake rate approaches 0 for these cells. Since autoinducers are continually synthesized according to the model, QS-mediated proteins are still being expressed. The cells at a higher density continue to take up the autoinducers as the distances between cells become smaller. Correspondingly, they experience lower autoinducer levels and lower metabolic activity, etc., and express less QS-mediated protein. Thus, in this configuration at higher values of β, the autoinducer-triggered protein expression would be lower for cells of higher density than for cells of lower density, which is not observed experimentally. Importantly, we include these simulations to illustrate both the model simplicity and the flexibility in the mathematical formalism ascribed to the simple adjustment of parameter β and the Gaussian uptake function.

With this model, which phenomenologically describes the natural phenomena, we have introduced a method of modeling quorum sensing that does not *a priori* require knowledge of the intrinsic gene and protein interactions that are necessary to communicate with other bacteria. Rather, the methodology depends only the distances between cells and simple parameters that provide the relative importance of those distances. That is, the flocking terms and similar density-dependent weights, as introduced here, serve to generalize the behavior of quorum sensing. We assumed here that QS marker protein expression in this uncoupled model formalism does not influence velocity. This keeps the ordinary differential equations simple enough to perform an asymptotic analysis (see Text S1) while encapsulating the dynamics of bacterial communication in tandem with chemotaxis.

### Coupled model.

The uncoupled model enabled the first mathematical representation of QS-mediated behavior as a form of flocking and was confirmed by asymptotic analyses (see Text S1). We now add a layer of complexity by creating a coupled model. By coupled, we mean the coupling of the velocity equation and the protein expression equation. Chemotaxis is the movement of bacteria triggered in response to a gradient in a concentration of particular molecular substances which are classified as either attractants or repellents. The changes in the concentrations of these substances activate a cascade of signal transduction mechanisms that affect motility (42).

Not much is known about the direct connections between the quorum sensing and chemotaxis systems, including for E. coli. Connections tend to describe the interactions or effects of one key protein in one system influencing the other system in some way. For example, AI-2 is a known chemoattractant for E. coli (43, 44), and the LsrB protein, which is responsible for AI-2 uptake, is necessary for chemotaxing to AI-2 (45). Thus, LsrB of the quorum-sensing system is integral to the chemotaxis system with regard to the movements toward AI-2. Also, upregulation in the motility and production of flagella was observed in E. coli *luxS* knockouts (46). That is, the regulatory systems in quorum sensing and chemotaxis consist of many genes, effector molecules, and proteins that contribute to the dynamical behaviors, as well as resultant activities (e.g., phosphorylation, induction, etc.) that are involved in these processes (47). Taking into account the various reactions and regulatory mechanisms of these systems can be complicated, as they are ill-defined, so instead, we aimed to generalize the behaviors of the quorum-sensing and chemotaxis systems again with the help of density-dependent weighted terms. Our revised model is represented in equations 6 to 9.

(6) $$\frac{dx_{i}}{\mathrm{dt}}=v_{i}$$

(7) dvidt=λ1N∑j=1Nk1(xi,xj)(vj−vi)+F0[υoe^s+(υ∞−υo)e^sψp(pi)−vi]

(8) $$\frac{dp_{i}}{\mathrm{dt}}=\frac{\lambda_{2}}{N}\overset{N}{\underset{j=1}{\sum }}k_{2}\left(x_{i},x_{j}\right)\left(p_{j}-p_{i}\right)+L_{0}\left(p_{\infty }-p_{i}\right)\psi_{A}\left(A_{i}\right)$$

(9) $$\frac{dA_{i}}{\mathrm{dt}}=\lambda_{3}\overset{N}{\underset{j=1}{\sum }}k_{3}\left(x_{i},x_{j}\right)r-k_{u}\left(x_{i}\right)A_{i}-k_{d}A_{i}$$

The velocity equation has been modified to be a function of protein expression. The strength of the source term for velocity is still governed by the constant *F*_0_, and *ê_s_* remains the unit direction vector. We have incorporated a logistic equation, Ψ*_p_*(*p_i_*), that takes values between 0 and 1 depending on the protein expression corresponding to that cell. Ψ*_p_*(*p_i_*) couples the protein expression to velocity, whereas Ψ*_A_*(*A_i_*) couples autoinducer activity to protein expression.

$$\begin{array}{l} \psi_{p}\left(p_{i}\right)=\frac{1}{{\left[1+e^{c_{0}}{}^{\left(c_{1}-c_{p}p_{i}\right)}\right]}^{\alpha_{1}}}\\ \psi_{A}\left(A_{i}\right)=\frac{1}{{\left[1+e^{c_{2}}{}^{\left(c_{3}-c_{A}A_{i}\right)}\right]}^{\alpha_{2}}} \end{array}$$

If Ψ*_P_* is 0 for all *t*’s, the source term reduces to *F*_0_(*v_o_ê_s_ – v_i_*). If Ψ*_P_* is 1 for all *t*’s, the source term becomes *F*_0_(*v*_∞_*ê_s_ – v_i_*). *v_o_* is the magnitude for the initial velocity. After the protein expression approaches a certain level, the cell's velocity increases, approaching *v*_∞_ if protein expression, *p_i_*, continues to increase. Protein degradation is assumed to be minimal. In the scenario when the source term for the protein expression equation is initially turned on [i.e., Ψ*_A_*(*A_i_*) takes on a nonzero value] but then turns off and remains off {Ψ*_A_*[*A_i_*(*t*)] is 0 for all *t*’s above *t*_threshold_, where *t*_threshold_ is the time when the autoinducer concentration drops below the threshold value}, we note that protein degradation should be taken into account for a more accurate analysis of long-term cell dynamics.

### Coupled model: discussion and application.

The 4*N* system of differential equations can be customized for specific quorum-sensing systems by using different threshold values, which are controlled by changing *α*_1_ and *α*_2_ (or, alternatively, *c_p_* and *c_A_*), modifying the scopes of influence (β_1_, β_2_, β_3_), customizing the uptake function in equation 9, and toggling the strength of each of the flocking and source terms (λ_1_, λ_2_, λ_3_, *F*_0_, *L*_0_). First, we use the same uptake function (5) as in the uncoupled model above.

**FIG 5:**
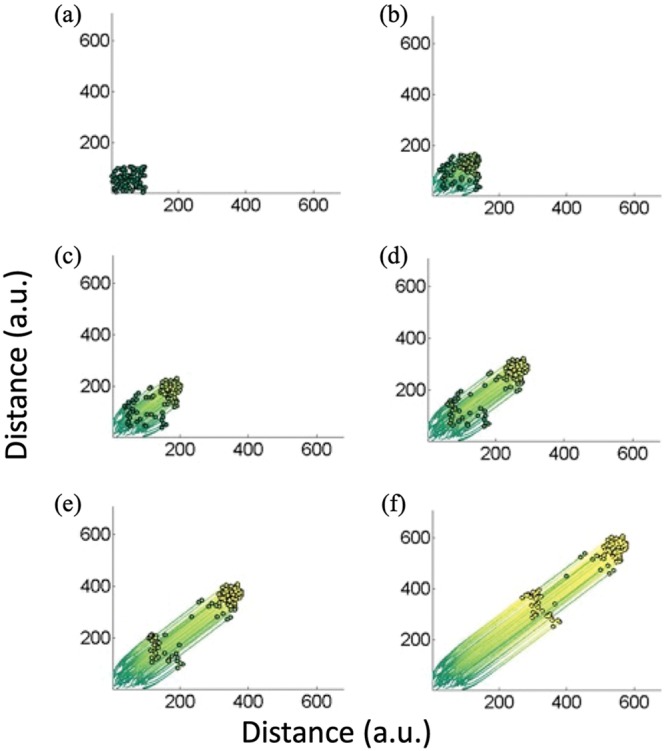
Simulations of a coupled model where velocity is linked to QS-mediated protein expression. (a through f) Population distributions as the population progresses in time. Cell populations were randomly distributed initially, as in Fig. 4. Two groups of cells emerged early on with different velocities. (b) The dense portion of cells at the upper right reaches a higher velocity. As it moves through panels c and d, it splits away from the rest of the group. At later times (e and f), the two groups seemingly remain as distinct groups. These phenomena were observed experimentally.

When the *α* values were equal (e.g., equal influence of velocity on protein expression and influence of autoinducer on protein expression), we observed an interesting dynamic within our cell population: two subgroups of cells had formed. Because this was not observed in the previous model under any condition, we conclude that this behavior was attributed to the coupling of quorum sensing and chemotaxis systems. This was an interesting finding that emerges only because of the dependence of velocity on the QS protein level. That is, in the uncoupled model, the cells exhibited trajectories in one single group, with only a portion of the group expressing more protein than the rest. Since quorum sensing does not influence velocity in that model, cells with higher expression would not move faster. The lengths of the trajectories in Fig. 2 and 3 were essentially similar. When we couple the model such that protein expression influences velocity, the portion with higher expression was seen to move faster, leaving the rest of the group behind. Not only did we observe that different cells had different velocities, in Fig. 5, we found the formation of two distinct subgroups. That is, this figure presents simulations starting from a randomized initial state (Fig. 5a, or similarly, the initial conditions in Fig. 4) and progresses in time (to Fig. 5f). That is, in Fig. 5b, a subpopulation of cells emerges from the group by moving to the upper right. This emerging subgroup of cells remains tightly formed as if in a flock. It is noteworthy that in this configuration, the QS-mediated protein expression is maintained. In essence, this also provides a means by which a subgroup of cells accelerates away from the others. This type of behavior, in general, is reminiscent of a bird flock.

The organization of cells into two groups, here, is consistent with experimental observations (35, 39). In chemotaxis experiments conducted over 50 years ago using capillary tubes (39), E. coli cells were placed at one end, and the chemoattractant (galactose or oxygen) was placed at the other end. E. coli moved toward galactose, as expected. What was unexpected was the appearance of distinct migrating bands. This is exactly analogous to the simulations in Fig. 5. The author, Adler (39), made an attempt to uncover a mechanism for this partitioning. He attributed the results to the varied consumption rates of nutrients and their placement at the distal end of the capillary tube. In one case, the first band of cells depleted oxygen while consuming part of the galactose, and the second band depleted the remaining galactose in what was remaining as an anaerobic environment. Interestingly, there was also a nonmotile fraction that stayed in place at the proximal end. To prove that this behavior was not attributed to individual cell heterogeneity, Adler scraped the cells from each of the two bands and repeated the experiment with those cells. Two groups of cells formed, yet again. He did not discuss or hypothesize how a subfraction of cells and not the entire population had emerged from the initial population in the first place.

Increased QS-dependent protein expression that is caused by the increased autoinducer locally near a high density of cells might lead to the emergence of a small group of cells transiting out of the larger group, especially if their velocity is linked to QS. This mechanism provides for the formation of two sets of cells during the process of chemotaxis, as described by Adler. Our model, for the first time using flocking formalisms, qualitatively reveals this emergent flocking behavior. Our simulations reinforce the idea that quorum-sensing systems, or analogous density-dependent mechanisms, are connected to the chemotaxis system of E. coli.

**FIG 6:**
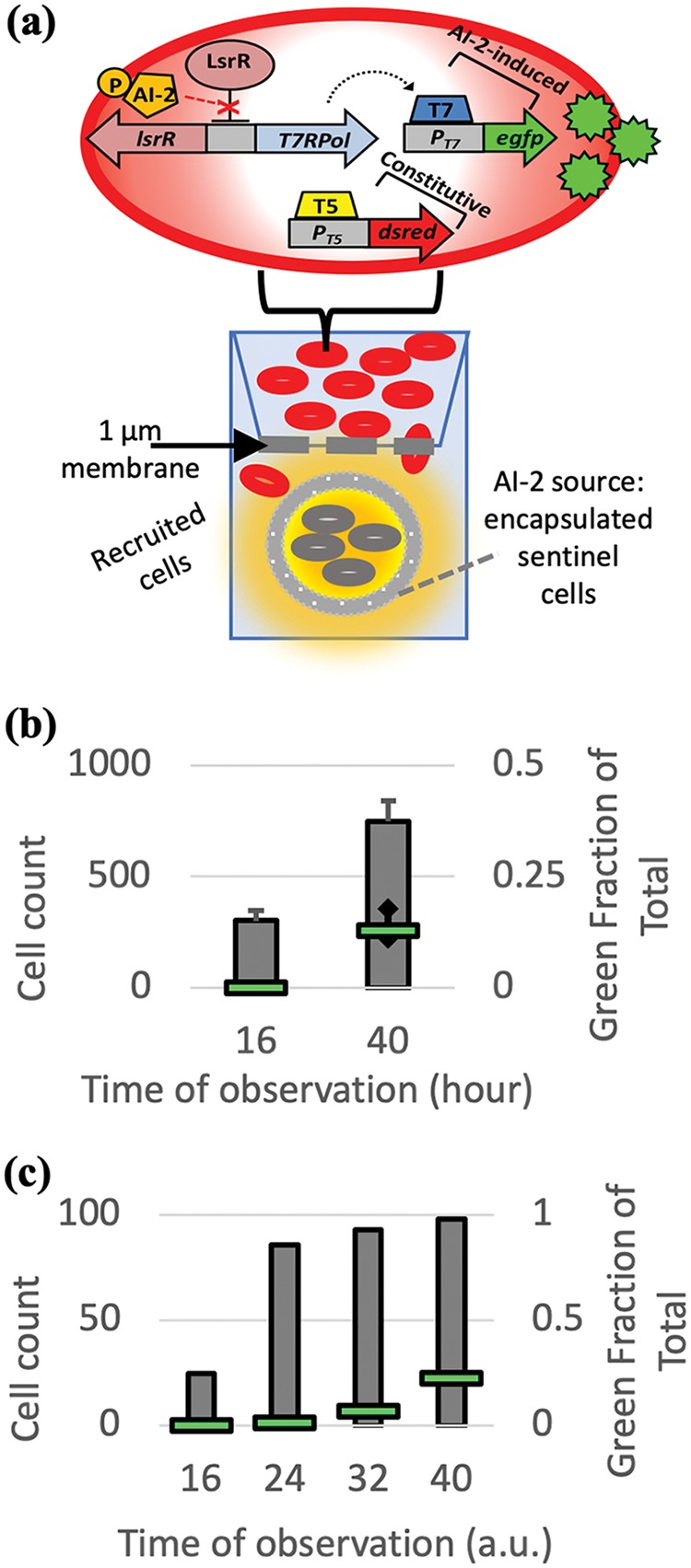
Encapsulated sentinel cells secrete AI-2 (orange) and draw recruiter cells through a membrane and into the bottom of a transwell apparatus. Data from the work of Servinsky et al. (35) are in panel b. (a) In the transwell experiments, initially, the recruitable cells are in the upper chamber and the sentinel cells are sequestered in alginate beads and placed in the lower chamber; the upper and lower chambers are separated by a membrane. The recruitable cells (Δ*luxS*) constitutively express DsRed and express enhanced green fluorescent protein (*egfp*) in the presence of AI-2 (via the *lsr* promoter). (b) The bar chart shows the number of cells in the lower chamber at hour 16 and hour 40. The fraction of cells expressing GFP is also indicated (horizontal green bars). The cells moved first, and subsequently, QS-mediated protein expression was actuated. (c) Our simulation results are qualitatively similar: the cell number in the lower chamber increases first, and the fraction of cells expressing protein increases later. Note that time is in arbitrary units (a.u.).

We next postulated thought experiments in which we interrogated the model seeking to observe the potential for QS-mediated microbial recognition. That is, what kind of response would we see when α_1_ was not equal to α_2_, reflecting scenarios where the threshold levels required for protein-coupled motility or autoinducer-coupled protein expression are varied relative to each other. Or, in what kind of experiment would we observe a scenario in which α_1_ is not equal to α_2_? We first respond to the latter question by describing the experiments conducted in a transwell apparatus by Servinsky et al. (35). There are two sets of cells in these experiments (Fig. 6). One set is “sentinel” cells that secrete AI-2 (Fig. 6a). These are placed in capsules that, in turn, are placed in the lower chamber of the transwell. The other set of cells, called “recruitable” cells, are genetically engineered to produce green fluorescent protein (GFP) when transcription is induced by AI-2 (48). Located in the upper chamber, these cells also constitutively express DsRed as a means of indicating their accumulation in the lower chamber of the transwell. These recruitable cells are engineered not to produce AI-2 (*lux* mutant) so that the only source of AI-2 was the sentinel cells located in the lower chamber. The transwell experiments therefore enabled us to track the movement of recruitable cells toward AI-2 (chemoattractant) by monitoring red fluorescence and, simultaneously, QS-dependent protein expression (AI-2-mediated expression of GFP). The experimental results (Fig. 6b) revealed cells (∼350) with DsRed, but not GFP, in the lower chamber at the 16-h time point. However, both GFP and DsRed had appeared by the 40-h time point (e.g., ∼15% green). The main conclusions in the work of Servinsky et al. (35) were, indeed, that a subset of cells moved toward a new locale that was, in turn, marked by the presence of autoinducer, AI-2, that had been secreted by distant sentinel cells. Also, because of the sequential nature of motility and QS-mediated gene expression, the work demonstrated further that the motility response was more sensitive than the quorum-sensing-triggered protein expression response with respect to AI-2. In such a scenario, α_1_ is less than α_2_. Such an observation was also noted in the work of Wu et al. (29), wherein AI-2 was generated on the surfaces of cancer cells so that payload-synthesizing bacteria could swim to those cells, synthesize a drug, and deliver it specifically to cancer cells and not healthy cells (devoid of surface-synthesized AI-2). There too, the AI-2 threshold for motility was lower than *lsr-*mediated gene expression.

Consequently, we modeled an analogous system in which chemotaxis was more sensitive than the (quorum-sensing-mediated) protein expression. This can be modeled by lowering the threshold of Ψ*_p_*(*p_i_*) (i.e., the value of *p_i_* for which the logistic function increases to 1) and raising the threshold of Ψ*_A_*(*A_i_*). That is, α_1_ was set to less than α_2_. Here, we use a modified uptake function in which the measure of density is the inverse of the sum of *k*_3_.

(10) $$k_{u}\left(x_{i}\right)={\overset{˜}{a}}_{1}\exp\left(\frac{-{\left\{\overset{˜}{a}\left[\frac{\gamma_{2}}{\sum_{j=1}^{N}k_{3}\left(x_{i},x_{j}\right)}\right]-\overset{˜}{b}\right\}}^{2}}{2\overset{˜}{c}}\right)+{\overset{˜}{a}}_{2}$$

In this simulation (Fig. 6c), we mimicked the upper and lower chambers of the transwell apparatus in the work of Servinsky et al. (35) by assigning a fixed distance from the origin of each cell trajectory to represent the membrane in the experiment. We recorded the number of cells and their protein expression level when they had swum past this membrane. We classified the cells that expressed greater than 0.7 arbitrary unit (AU) in our model as fully expressing GFP. Figure 6c shows the increasing cell count in the lower chamber of our simulation as well as the increasing fraction of cells fully expressing GFP. More specifically, the number of cells that had migrated to the lower chamber had increased dramatically by the second time point (24 AU), and the GFP-expressing fraction increased appreciably only after the third time point (32 AU). We qualitatively compared these simulations to the results of Servinsky et al. (35), shown in Fig. 6b, and we observe similar results: as time passed, the cell count increased but GFP was not expressed until later. By the incorporation of flocking interactions and by accommodating signal-mediated sensitivity for chemotaxis and QS-mediated gene expression, our flocking model for the first time was able to demonstrate the potential for a subset of bacterial cells to seek out new environments and, upon entrée into the new locale, to exhibit classic QS behavior. We know of no other modeling efforts that have described these phenomena.

## DISCUSSION

We have introduced a generalized approach to modeling quorum sensing as an extension of flocking, an attribute normally ascribed to large animals with multiple sensing domains and computational foci, such as a brain. Yet, for relatively simple prokaryotic bacteria, the same population-based behavior can be described. Interestingly, the suggestion that bacterial phenomena mimic the behavior of higher-order species was made by Adler over 50 years ago.

We presented both an uncoupled and a coupled model to represent a quorum-sensing, chemotaxing group of bacteria. The model does not require mechanistic knowledge behind the specific signal transduction and regulatory mechanisms involved in either quorum sensing or chemotaxis but is based on phenomenological observations. Also, the Cucker-Smale flocking terms, the source terms, and the density-dependent weighted terms for autoinducer synthesis and uptake generalize the complex signaling processes at play.

We used the uncoupled model in order to perform asymptotic analysis and to understand the dynamics of the model. We also developed an understanding of parameter values (e.g., β) that enable qualitative agreement with the natural phenomena. We then used the coupled model to explore the results of autoinducer-triggered protein expression on the velocity of the cell. Simulations showed the formation of two groups of motile cells upon recognition of a chemoattractant. To the best of our knowledge, this behavior has not been described mathematically even though it was first observed decades ago. When our system of differential equations was decoupled so that chemotaxis was not dependent on quorum sensing [i.e., Ψ*_p_*(*p_i_*) = 0], the cells remained in one group. The emergence of two groups was due to autoinducer-triggered protein expression influencing the cell velocity. This suggests the possibility that chemotaxis is influenced directly by QS or by another, as-yet-not-modeled regulatory process that is also density-dependent. Future experiments that attempt to uncover such a link might be warranted, for example. Such a fortuitous observation sheds light on one of the benefits that stems from higher-order-population-based models. When we simulated a system in which the chemotactic response was more sensitive than the quorum-sensing-triggered protein expression, we obtained trends similar to those produced in transwell experiments (35). We also observed how subpopulations of motile cells emerged from the rest, creating discrete flocks of uniform behavior. These too might suggest novel paths of experimental inquiry.

Future modeling directions might include stochastic elements that account for random variations observed in movement and in behavior. Such stochastic input, as alluded to in the introduction, would allow for individuality or a small subgroup of coordinated behavior to transition the entire population in one direction or another, such a behavior not prescribed. This would also enable random processes as applied to movement, velocity, protein expression, and autoinducer concentration to affect the system, and in turn, it would facilitate a closer representation of true bacterial chemotaxis and quorum-sensing interactions that are no doubt dependent on the integration of many cues.

## MATERIALS AND METHODS

In this paper, data were obtained from the references indicated. Simulations were performed using ode45 in MATLAB (49), with relative and absolute tolerances set at 10^–9^. Visualizations in this paper were performed using MATLAB and Microsoft Excel (50). Projecting a color gradient onto a line using interpolation was inspired by the cline MATLAB function.

### Data availability.

We will gladly share MATLAB code.
