# Supplementary material for: Bacteria Floc, but Do They Flock? Insights from Population Interaction Models of Quorum Sensing
Source: mBio. 2019 May 28;10(3):e00972-19. doi: 10.1128/mBio.00972-19 (PMC6538791; doi:10.1128/mBio.00972-19)
Supplement: TEXT S1 [file mBio.00972-19-s0001.docx]

Supplementary - Propositions


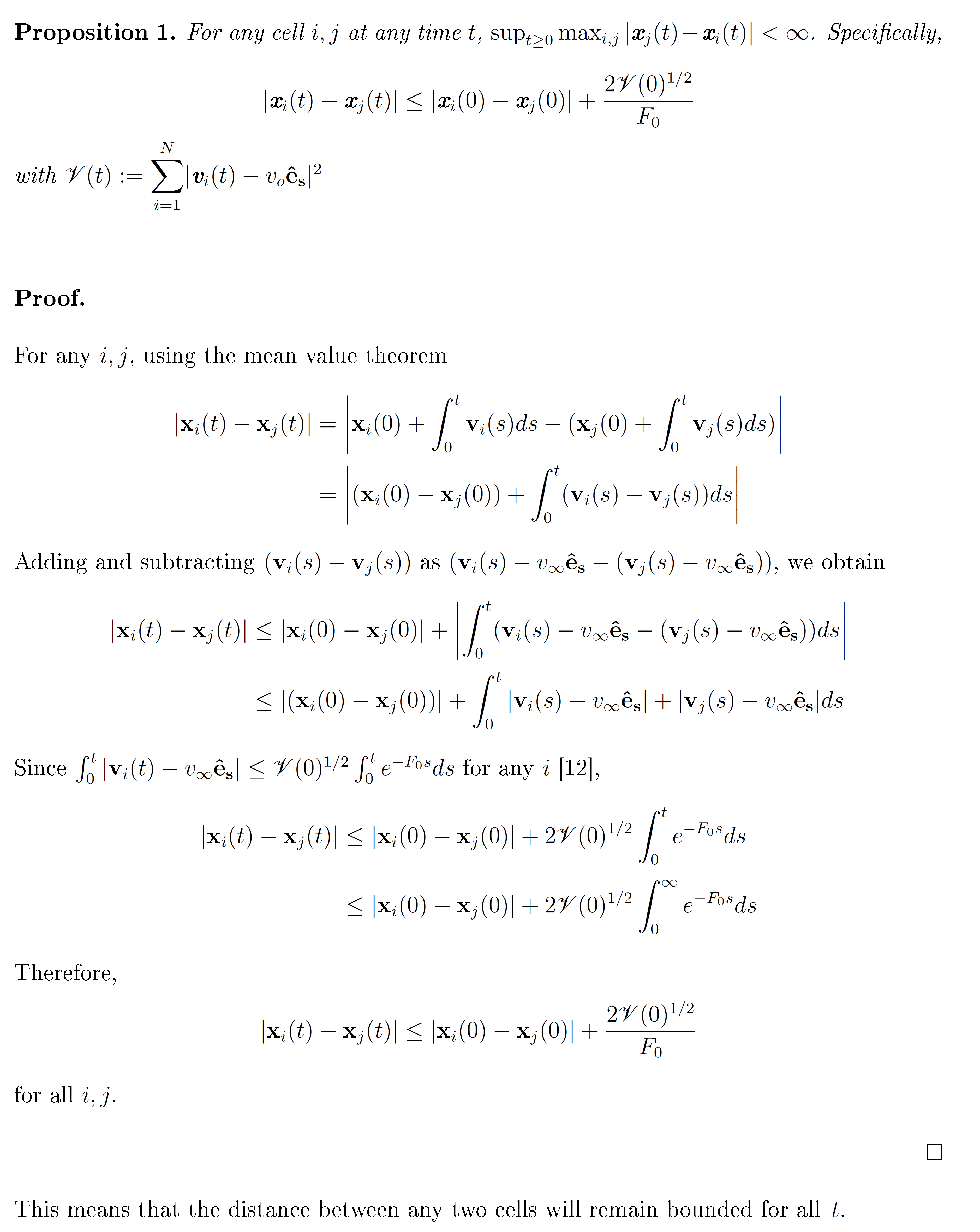


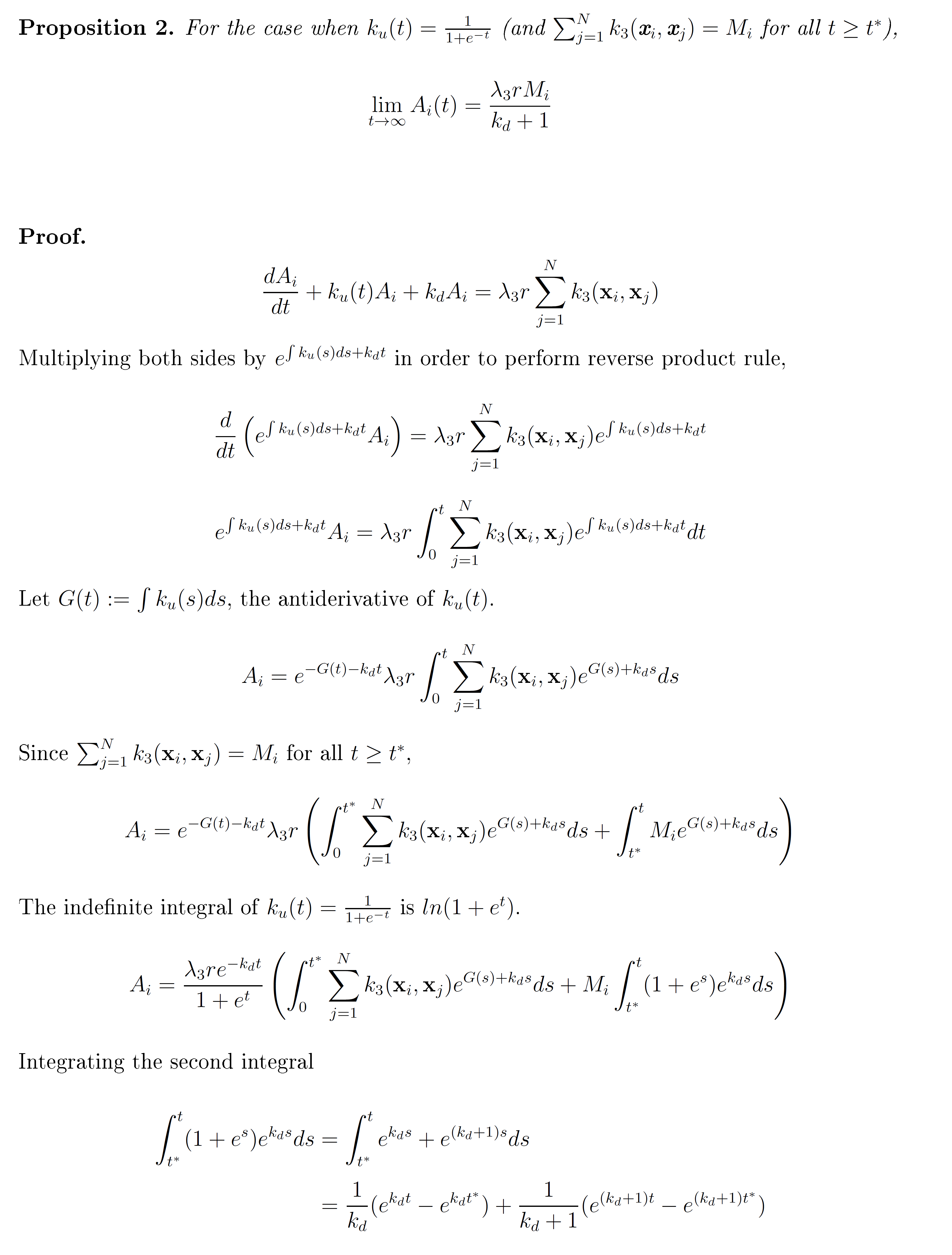


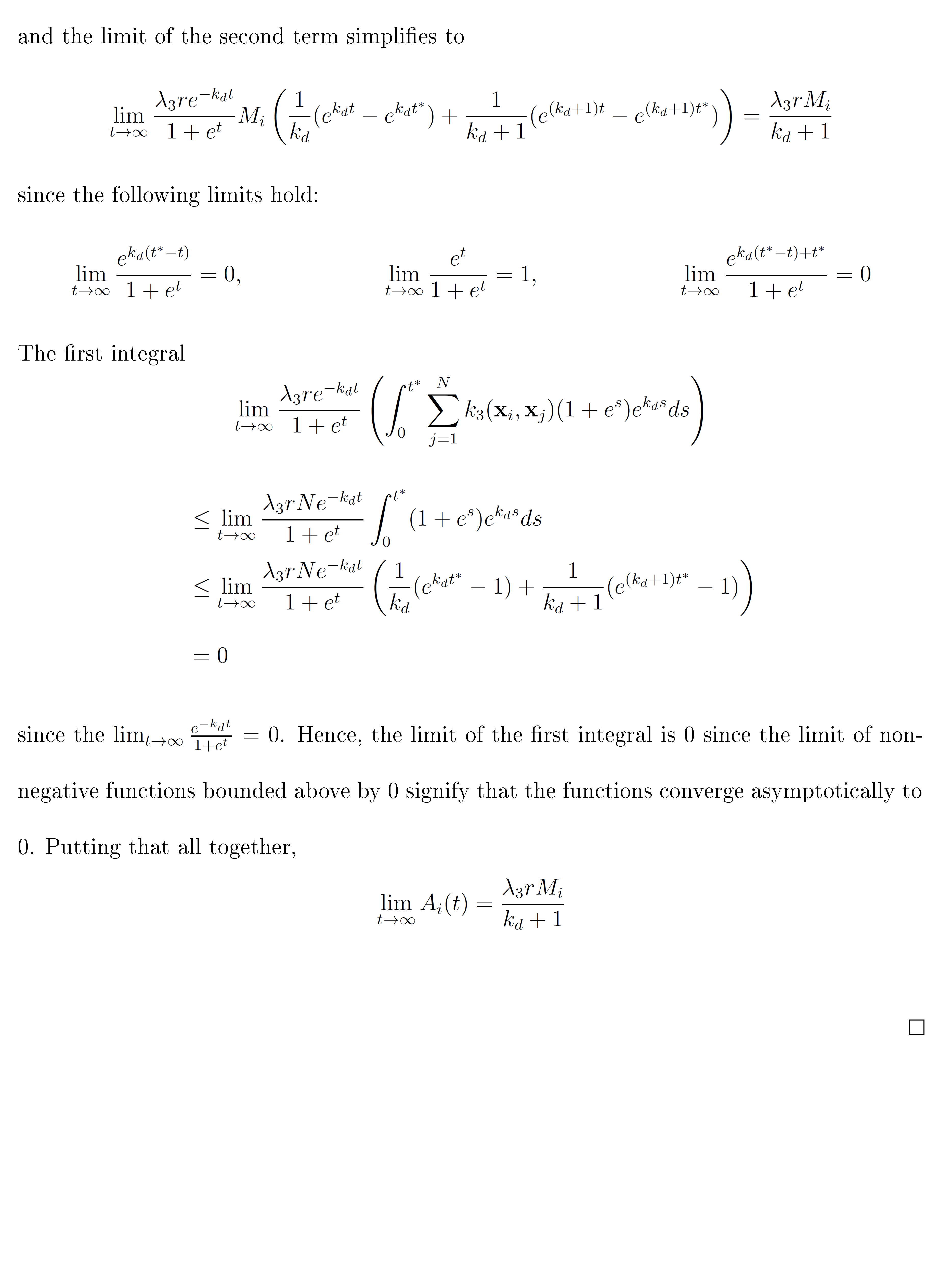


*Uncoupled Model: Asymptotic analysis*

In this section, we show that the system represented by equations (1)-(4) satisfy the

above definition of flocking. We also apply asymptotic analysis to our system of differential equations in order to understand the overall dynamics of the model without the use of simulations. We have the following properties for our weighted model. More details on these properties can be found in Ha and Levy([18](#_ENREF_18)) or in Ueda (PhD Dissertation).

Properties (i) and (ii) prove that the weighted model satisfies the conditions of flocking. Property (i) states that, following the assumption that velocity of each cell is the weighted average of the velocities of cells surrounding it, the velocity of each cell approaches the value voê_s_. vavg(t) asymptotically approaches the same value as voês. So, the variation in the velocities with respect to the average velocity approaches 0 asymptotically. That is, there is little to no variance in velocity as time progresses. Property (ii) states that for any time t, the position of any cell is bounded by a constant. This constant is determined by the initial positions and velocities of the cells (which we assume to be finite) and F0.

Property (iii) states a similar result to Property (i) when Ψ_A_ = 1. If Ψ_A_ = 0 for all t, then $\lim_{t\to\infty} p_{i}(t)=\frac{1}{N}\sum_{j=1}^{N} p_{j}(t)$ for β < 1/2.([32](#_ENREF_32)) To put this in the context of *Vibrio fischeri*, each of the cells will collectively express bioluminescence at some average level.

To perform asymptotic analysis on Equation (4), we first make assumptions on the form of ku(xi). Since the form of Equation (5) causes difficulties in solving the linear differential equation, we focus on the simple case of $k_{u}\left( t \right)=\frac{1}{1+e^{-t}}$. This applies to scenarios in which the rate of uptake is initially low and increases to a stable or maximum uptake rate over time. In other words, this can be observed in scenarios in which the cell density increases up to a certain point and maintains that density. (Note that this logistic function can be generalized but we work with this simple case in order to present a proof less cluttered.) The limit reveals the intuitive result that the surrounding concentration of autoinducers around cell i is ultimately determined by synthesis of autoinducers, the surrounding density of cells, and the rate at which autoinducers are left behind during swimming or degraded.
